# Indirect niche construction: A coffee pest facilitates its own ant natural enemies

**DOI:** 10.1002/ecy.70463

**Published:** 2026-07-24

**Authors:** Jonathan R. Morris

**Affiliations:** ^1^ School for Environment and Sustainability University of Michigan Ann Arbor Michigan USA; ^2^ Laboratorio Nacional de Ciencias de la Sostenibilidad, Instituto de Ecología Universidad Nacional Autónoma de México Mexico City Mexico

**Keywords:** agroecosystems, ants, coffee, natural enemies, natural pest control, niche construction

The ubiquity of niche construction in shaping ecosystems and their interactions is increasingly recognized by ecologists (Laland et al., 2017; Matthews et al., 2014; Odling‐Smee et al., 2013). We now understand that many organisms modify their environments in a manner that has adaptive implications for individuals and in turn, may impact the dynamics of those species' populations, both on ecological and evolutionary time scales (Laland et al., 2016). What is less well understood is how niche construction by one species impacts the fitness and ecological dynamics of other species (Kylafis & Loreau, 2011; Vandermeer, 2008). In predator–prey and consumer–resource interactions, there are salient examples where predators or consumers modify their niche to enhance the capture or consumption of their resource (Laland et al., 2017; Matthews et al., 2014). As examples, spiders construct webs to catch prey and leaf cutter ants cultivate fungal symbionts in their nests that are consumed as food. Similar top‐down, indirect effects mediated by niche construction are likely common in ecosystems. However, effects in the opposite direction, where a prey's niche construction has a bottom‐up positive impact on a predator should happen much less frequently, due to the obvious negative fitness consequences for the prey and the potentially destabilizing impacts on predator–prey dynamics. When predators are facilitated by a prey resource, overexploitation could result in unstable predator–prey cycles (Arditi & Berryman, 1991; Luck, 1990; Vuorinen et al., 2021). If predators are broad generalists, however, the positive indirect impacts of prey niche construction may be diffuse enough to allow these interactions to persist.

In agroecosystems, natural pest control can help to regulate pests when natural enemies are promoted by conserving habitat (Bianchi et al., 2006). Ants are common generalist natural enemies in agroecosystems and contribute to pest control in many agricultural systems (Anjos et al., 2022; Offenberg, 2015; Sankovitz et al., 2024). In coffee agroforests, ants play a key role in regulating the worst insect pest of coffee, the coffee berry borer (*Hypothenemus hampei*) (Morris et al., 2018; Philpott & Armbrecht, 2006). This small beetle specializes on the fruits of coffee, boring into them to lay their eggs (Figure 1a,b), where larvae and new adults consume the endosperm until reproductive females emerge to colonize new fruits (Vega et al., 2009). The borers thus construct their own refuge, spending the majority of their lifecycle inside bored coffee fruits. While some species of larger ants that forage on coffee help to reduce the colonization rates of the coffee borer (Gonthier et al., 2013; Morris et al., 2015), smaller ant species can enter borer galleries in fruits to remove adults and immature individuals (Constantino‐Chuaire et al., 2022; Larsen & Philpott, 2010; Moreno‐Ramírez et al., 2025; Morris & Perfecto, 2016). Many of these smaller twig and cavity nesting ant species (Figure 1c–e) could also potentially take advantage of bored out fruits as habitat.

**FIGURE 1:**
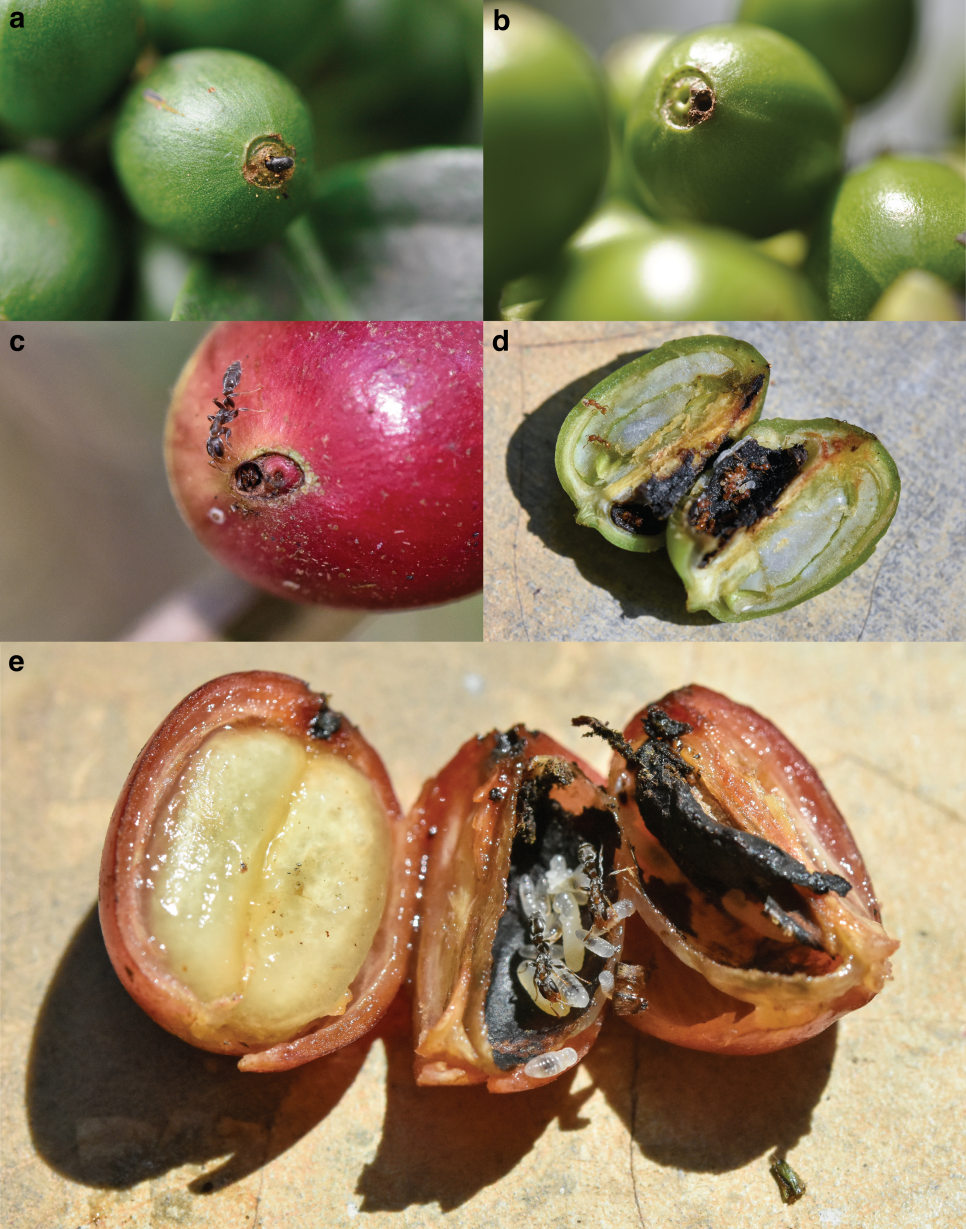
Natural history of ant‐borer‐coffee fruit interactions. Photos depict (a) a coffee berry borer (*Hypothenemus hampei*) boring into a coffee fruit, (b) the perforation damage of the fruit caused by the borer, (c) a *Pseudomyrmex elongatus* worker next to a borer gallery with another ant worker inside, (d) a *Wasmannia auropunctata* nest, and (e) a *P. elongatus* nest, both with brood inside bored coffee fruits. Photos taken in 2014 (a, b) and in 2025 (c–e) by Jonathan R. Morris at Finca Irlanda in Chiapas, Mexico.

While working on an organic, high‐shade coffee farm in Chiapas, Mexico, I observed just this behavior. In September and October of 2016, I collected hundreds of green coffee (*Coffea arabica*) fruits with evidence of coffee berry borer damage to extract borers for a laboratory experiment at our field station in Finca Irlanda, published with Ivette Perfecto (Morris & Perfecto, 2022). Upon dissecting these fruits, I discovered on occasion, ants inside of them, and documented at least seven different species (Table 1), several of which were later photographed in November 2025. The novelty of this observation is that five of the seven ant species showed clear evidence of using bored fruits for reproduction, with either brood (eggs, larvae, or pupae), queen, or alate (reproductive) individuals present inside of the coffee fruits (Table 1 and Figure 1d,e). It appears that these species gain a fitness advantage from the damage of the coffee borer, indirectly benefiting from the niche construction of their prey.

**TABLE 1:** List of ant species documented inside coffee fruits in this study and their role in coffee berry borer (*Hypothenemus hampei*) control.

Ant species	Life stage/caste found in fruits	Role in coffee berry borer pest control	Literature with experimental evidence
*Azteca* sp.	Workers, majors	Unknown for this species, but congeneric *A. sericeasur* attacks adult borers and reduces colonization	Perfecto and Vandermeer (2006), Pardee and Philpott (2011), Philpott et al. (2012), Gonthier et al. (2013), Jiménez‐Soto et al. (2013, 2019), Morris et al. (2015), Rivera‐Salinas et al. (2018), Morris and Perfecto (2022), Cowal et al. (2023)
*Nesomyrmex echinatinodis*	Brood, workers, alates, queen	Unknown	NA
*Pheidole* sp.	Workers, majors	Unknown for this species, but other *Pheidole* spp. attack adult borers and reduce colonization	Varón et al. (2004), Gonthier et al. (2013), Jiménez‐Soto et al. (2013)
*Procryptocerus hylaeus*	Brood, workers	Known to reduce borer colonization	Philpott et al. (2012)
*Pseudomyrmex elongatus*	Brood, workers, alates	*Pseudomyrmex* ants in general are known to reduce the densities of borers inside fruits and overall borer colonization	Larsen and Philpott (2010), Philpott et al. (2012), Gonthier et al. (2013)
*Tapinoma* sp.	Brood, workers, queen	Some *Tapinoma* ants are known to reduce borer colonization	Gonthier et al. (2013), Onishi et al. (2017)
*Wasmannia auropunctata*	Brood, workers	Known to reduce borer colonization and predate immature borer life stages	Gonthier et al. (2013), Morris and Perfecto (2016), Morris and Perfecto (2022)

*Note*: Ant life stages and castes are listed as well as the known role of each species in coffee berry borer biological control with supporting literature references.

This observation, however, raises important additional questions about the natural history of this behavior in the ants. For example, while it is known that smaller species of ants can enter fruits to reduce borer densities, it is not clear if the ants observed in this study are actually consuming borers or simply removing them to gain access to the niche space of the hollowed‐out coffee fruits. These details are difficult to discern directly in the field, but other laboratory and field research suggests that ants that enter fruits also consume borer immature (Constantino‐Chuaire et al., 2022; Moreno‐Ramírez et al., 2025; Morris & Perfecto, 2016) and adult life stages (Larsen & Philpott, 2010). It is also not clear if, or to what extent, the ants themselves further manipulate the fruits to enhance this refuge space. I observed that in most cases, when ants were present, the cavities inside bored fruits were partly cleaned out, which is not typically seen when only the berry borer is present. It is likely that ants, upon entering bored fruits, remove the borer waste and decayed endosperm that is found in heavily infested fruits to create a better suited refuge for ant reproduction. I have also separately observed in the field that some ants found entering bored coffee fruits appear to slightly widen the gallery hole originally created by borers. In this sense, the ants may take advantage of the niche provided by borers and then enhance those modifications, in a kind of synergistic niche construction. Similar modified niche construction behavior by ants has been documented in several oak‐gall colonizing ant species in Europe, where ants will enter abandoned Cynipid wasp galls on oak trees and use these woody structures to nest, sometimes further excavating the cavities inside and partially closing the nest entrance hole (Giannetti et al., 2019, 2022; Torossian, 1967). However, it is unclear in these systems if the ants also predate the gall‐inducing wasps that they benefit from, since wasp emergence is most likely required for initial ant entry into the galls. Finally, with the two ant species encountered in fruits that did not exhibit evidence of reproduction, it is possible that in some circumstances ants simply use the hollowed‐out fruits as a refuge from microclimate extremes or to escape their own natural enemies, such as parasitoid phorid flies, which are known to attack *Azteca* ants in this system (Pardee & Philpott, 2011).

Overall, while this behavior may not always occur when ants and coffee berry borers interact and is limited only to certain times of the year when coffee fruits are sufficiently large and borers have had enough time to do their damage, the fact that several species of ants were observed reproducing and seeking refuge in fruits suggests that this is not a one‐off event. Moreover, the reproductive benefit received by these ants suggests that this prey species is facilitating the populations of its own natural enemies, bolstering the presence of these species directly on coffee to potentially enhance regulation of future colonizing borers or borer damage in other nearby fruits.

More broadly, I present a fascinating example of what I call “bottom‐up indirect niche construction,” where the niche modifications of a prey or resource species indirectly enhance the fitness of its own predator or consumer. While there are sure to be other novel examples of this unexpected interaction in nature, it is not likely to be common, considering the negative impact on the niche constructing species and the possible destabilizing dynamical effect it could have if it leads to overexploitation (Arditi & Berryman, 1991; Luck, 1990; Vuorinen et al., 2021). Perhaps only if limited to certain spatiotemporal contexts, or when predators or consumers are broad generalists, can this type of interaction be sustained. Furthermore, in agroecosystems, where natural pest control is a goal, it should be remembered that natural enemies benefit from their prey, and perhaps tolerating low levels of pests, and their potentially beneficial niche modifications, may help to maintain natural enemy communities and their complex interactions in the long term so that a kind of autonomous pest control can be achieved (Lewis et al., 1997; Morales & Perfecto, 2000; Vandermeer et al., 2010).

## CONFLICT OF INTEREST STATEMENT

The author declare no conflicts of interest.
